# Unwanted Loneliness and Mental Health: Current State of Knowledge

**DOI:** 10.1192/j.eurpsy.2025.1475

**Published:** 2025-08-26

**Authors:** R. Fernández-Fernández, Á. Izquierdo de la Puente, P. del Sol Calderón, M. Vizcaíno da Silva, M. Sánchez-Duque, D. Gómez-Olmeda

**Affiliations:** 1 Hospital Universitario Infanta Cristina, Parla; 2 Hospital Universitario Puerta de Hierro Majadahonda, Majadahonda; 3 Centro de Salud San Carlos, San Lorenzo de El Escorial, Spain

## Abstract

**Introduction:**

Loneliness and social isolation are frequently associated with mental health problems. In Spain, the term ‘unwanted loneliness’ has gained particular importance in recent years, referring to an involuntary insufficiency in social relationships. According to recent studies, this affects an estimated 13.4% of the Spanish population (Jiménez Rodríguez. Rev Esp Salud Publica 2024; 98).

**Objectives:**

To conduct a review of the studies that address loneliness and social isolation

**Methods:**

We searched in PubMed using the following terms: ‘((unwanted loneliness[Title/Abstract]) OR (loneliness[Title/Abstract]) OR (social isolation[Title/Abstract])) AND (mental health[Title/Abstract])’, limiting the search to the last 30 years.

**Results:**

We found 5,472 articles on the subject, of which 4,373 were published in recent years, confirming the growing interest in this issue. The countries with the most publications on this topic were England (1,733 results), the United States (1,561 results), and Switzerland (894 results). Although we did not focus on this aspect, it is worth noting that the most frequently used keyword was ‘COVID-19,’ appearing a total of 1,229 times, which may partially explain the increase in publications over the last year.

Upon reviewing the content of the articles, we observed that many focus on demographic factors. For example, living with a romantic or sexual partner has been consistently identified as a protective factor against loneliness (Currin et al. Curr Psychol 2022; Online publication), and we also found significant associations between loneliness and being single, separated, or divorced (Ibáñez-Del Valle et al. Int J Environ Res Public Health 2022; 19:16622).

The association of this issue with social inequalities has also been highlighted (Martín Roncero et al. Gac Sanit 2021; 35:432-437). We observed a potential gender bias, with findings indicating a higher risk in women for the perception of loneliness and the evaluation of social relationships (Pavlidis et al. Aging Ment Health 2023; 27:1313-1321). Other studies have found that higher population density reduces social isolation in areas with a high proportion of people of the same race or ethnicity but increases it in areas with fewer people of the same ethnicity (You et al. Inquiry 2024; 61:469580241273127). Sexual orientation-related factors also appear to be significant: internalized homonegativity has been associated with loneliness, where it is noted that accepting and integrating a gay or lesbian identity seems particularly important for younger, non-gay-identified individuals (Berg et al. J Gay Lesbian Ment Health 2015; 19:285-302).

**Conclusions:**

Unwanted loneliness is a complex and highly significant phenomenon, with a demonstrated association with poorer overall and mental health (Martín Roncero et al. Gac Sanit 2021; 35:432-437). This issue should be studied not only from the lens of mental health but also from a sociological perspective.

**Disclosure of Interest:**

None Declared

